# Synthesis Procedure of Highly Densely Packed Carbon Nanotube Forests on TiN

**DOI:** 10.3390/nano9040571

**Published:** 2019-04-08

**Authors:** Teresa Campo, Sergio Pinilla, Santos Gálvez, José María Sanz, Francisco Márquez, Carmen Morant

**Affiliations:** 1 Laboratory of Coatings and Nanostructures, Department of Applied Physics, Universidad Autónoma de Madrid (UAM), Cantoblanco, 28049 Madrid, Spain; mariterecampo@gmail.com (T.C.); sergio.pinilla@uam.es (S.P.); sgalvez@cab.inta-csic.es (S.G.); josem.sanz@uam.es (J.M.S.); 2 Instituto Nicolás Cabrera, Universidad Autónoma de Madrid, 28049 Madrid, Spain; 3 Nanomaterials Research Group, Department of Chemistry, Universidad Ana G. Méndez-Gurabo Campus, 189 St. Rd. km 3.3, Gurabo, PR 00778, USA

**Keywords:** TiN, SWNT, AC-CVD, Mo-Co catalysts, field emission

## Abstract

The goal of this research was to obtain high-density single-walled carbon nanotube forests (SWNTs) on conductive substrates for different applications, including field emission. For this, dip-coating was chosen as the catalyst deposition method, to subsequently grow SWNTs by Alcohol Catalytic Chemical Vapor Deposition (AC-CVD). Si (100) was chosen as the substrate, which was then coated with a TiN thin film. By sputtering with Ar, it was possible to generate alternating TiN and Si lanes, with a different wettability and, therefore, a different affinity for the catalysts. As a result, the Mo-Co catalyst was mainly deposited on TiN and not on sputtered-Si, which allowed the selective growth of SWNT forests on the TiN conductive surfaces. These as-synthesized SWNTs were used for field emission measurements in a high vacuum chamber.

## 1. Introduction

Carbon nanotubes (CNTs), can be considered cylinders of graphene rolled up into one or multiple layers (denoted single-walled, SWNTs, or multi-walled, MWNTs, carbon nanotubes), giving rise to materials with a variety of diameters and extraordinary properties. Although they began to be known as of 1991 thanks to Iijima [1], these materials had been synthesized much earlier [2,3]. CNTs are among the most fascinating materials that have been synthesized, and have extraordinary physical properties that include a high aspect ratio [4], high mechanical strength [5], thermal stability [6,7], and very high electrical conductivity similar to that of copper [8]. These properties make these materials of interest in applications in optics, the development of electrical circuits and transistors, field emission, mechanical engineering, pollution control, purification and filtering, sensors, energy storage, biomedical science, and even as a structural reinforcement in advanced composites [4,5,8,9,10,11,12,13].

Although the synthesis of CNTs is considered a well-established technology, hundreds of papers are still published every year with the aim of improving these processes under different experimental conditions. In these studies it is established that the structure and morphology of the nanotubes will determine for what kind of applications they will be useful. Most industrial synthesis procedures are focused on the obtaining of bulk CNTs for applications in the development of composites or thin films, with unorganized architectures, which obviously limits their use in many other applications [14]. In addition, CNTs of small diameter, and especially those with a single wall (SWNTs), have greater economic value, derived from their improved physical properties, greater field of applications, and greater difficulty of synthesis [14].

In recent years, much of the research in these materials has focused on the growth of dense SWNT forests on different substrates [15,16,17,18,19,20]. This architecture allows the use of these materials in different applications, including field emission, energy storage, nanoelectronics, or sensing. Although different methods of synthesis can be used, chemical vapor deposition (CVD) is considered the method that allows the greatest control of the process, leading to better results [21,22]. In fact, when the goal is to carry out patterned growth of vertically aligned SWNTs (VA-SWNTs), alcohol catalytic CVD (AC-CVD) is one of the techniques of choice. Most research related to these architectures is based on the use of insulating substrates [23,24], which facilitate the nanoparticulate disposition of the catalyst, allowing the growth of densely packed SWNTs. Traditionally, carbon nanotube forests are grown using metal catalysts deposited on oxide supports. Among these catalysts, Ni, Co, Mo, Fe, or a combination of them stand out, while the oxide supports mainly include Al_2_O_3_ or SiO_2_. The choice of insulating supports and possible catalysts has its justification in the surface energies of the oxides, which are generally lower than those of metals. In this sense, the supports most used in this growth, namely alumina or silica, provide strong interaction with some metals, which helps stabilize the small solid particles of catalyst [24]. However, for some of the applications it is essential to use conductive substrates, specifically for field emission or nanoelectronics, providing an electrically conductive path through the nanotube support [25,26,27,28]. Depending on the deposition route, the catalyst can be incorporated as a metallic species (for example, by sputtering), or as an oxide (in wet impregnation processes). In this last case, the pre-treatment of the substrate-catalyst under annealing in a reducing atmosphere induces the transformation of the oxidized species into metallic nanoparticles whose properties and size allow the growth of nanotubes. In fact, the formation of metallic nanoparticles of the catalyst is favored by the insulating nature of the support, giving rise to catalyst nanoparticles whose dimensions are critical for the growth of the nanotubes.

In recent years, many options have been tested to synthesize high-quality carbon nanotube forests on conductive substrates. Some of these investigations require multiple stages of preparation of substrates, gradients of composition, and complicated processes of catalyst formation, which even include the presence of traces of water and oxygen [29,30] as growth-directing agents, making their scalability difficult. Other preparation strategies consist of the use of barrier layers, such as alumina or Ti, which lies between a layer of catalyst (Ni or Fe) in the upper part and a metallic substrate in the lower part [31,32,33]. The barrier layer prevents intermetallic diffusion or even catalyst poisoning. Some metallic substrates grow carbon nanotubes directly without the need for a catalyst, and after some surface treatments, including acid etching and others [34]. However, all these procedures require multiple stages and the processes are not simple.

The objective of this research was to obtain carbon nanotube forests on a conductive substrate (TiN), which allows its use in a specific application, in our case field emission. TiN is a suitable material because it adheres to the Si substrate, allowing the material on which the nanotube growth takes place to be totally conductive. A TiN thin film was deposited by sputtering on a Si substrate. The deposition of the catalyst, a mixture of Mo-Co, was carried out by dip-coating, a wet chemical method that, during the experimental procedure, allows us to control the incorporation of the catalyst in specific areas on the substrate. In this way, the different wettability properties shown by the TiN thin film and the Si substrate on which it is deposited allow efficient distribution of the catalyst during the impregnation process. As a result, dense single-walled carbon nanotube forests have been selectively grown in areas with TiN, whereas in regions of Si the growth was inhibited.

## 2. Materials and Methods

### 2.1. Preparation of Substrates

P-type silicon wafers (100) from El-CAT Inc. (Waldwick, NJ, USA) used as substrates, were previously cleaned with isopropyl alcohol in an ultrasound bath for 10 min, and dried in an oven at 343 K for 1 h. Si substrates were coated with a thin TiN film using a Dual Ion Beam Sputtering (DIBS) Deposition System. A TiN target (99.5% pure, Kurt J. Lesker, Clairton, PA, USA) was evaporated by Ar^+^-sputtering, using a Kaufman-type ion source at an accelerating voltage of 700 V at base pressure of 10^−5^ mbar. Si substrates are in a sample holder that is approximately 10 cm from the target of TiN, and that is kept thermostated, maintaining a constant rotation so that the deposition is as homogeneous as possible. This system is complemented by an end-Hall type ion source, used to assist the deposition with nitrogen, which is simultaneously operated at 100 V, obtaining in this way a deposit film close to the stoichiometric TiN. Flow rates of nitrogen gas (99.999% purity) and argon gas (99.999% purity) were identical and controlled by mass-flow controllers. The deposition temperature was 493 K, with a deposition time of 4 h [35]. The film thickness (200 nm) was monitored with a quartz oscillator, and subsequently confirmed by SEM.

To obtain samples composed of alternating regions of TiN and Si, Ar^+^ sputtering was used to remove, in selected areas, the previously deposited TiN film. This sputtering was made by using micrometric size masks. The mask was placed on the surface of previously synthesized samples (with TiN grown on Si with its natural oxide), and then subjected to sputtering with Ar^+^. As a result, the areas of the sample exposed to the Ar beam experienced sputtering, giving rise to areas without TiN, while the parts of the sample covered by the mask were protected from attack, maintaining the deposition of TiN on the surface. For this process, the Kaufman-type Ar source previously used was placed perpendicular to the sample, using an acceleration voltage of 850 V, and a total sputtering time of 7 min, which allowed us to eliminate the layer of TiN and expose the unoxidized support, namely sputtered-Si.

**Figure 1:**
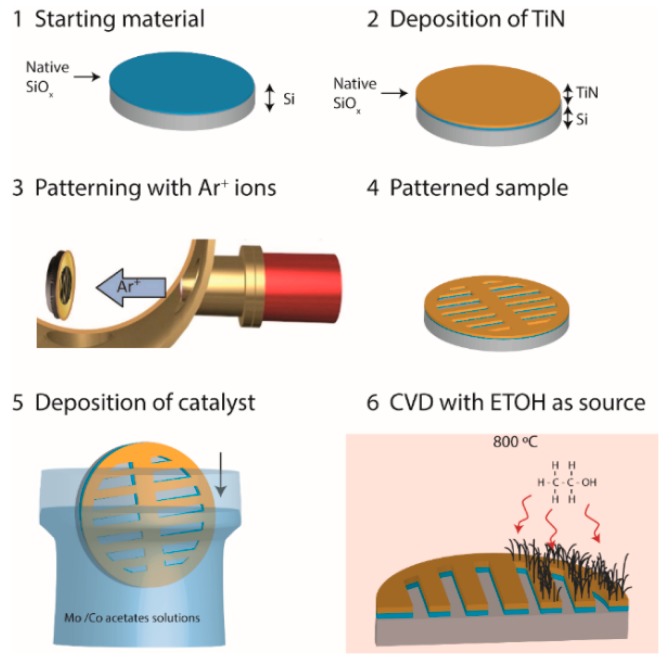
Scheme of the different steps (**1**–**6**) for the preparation process of the TiN/sputtered-Si substrates, and the subsequent growth of SWNTs.

The deposition of the catalyst on the substrates whose preparation has been described above is detailed elsewhere [36]. In brief, Mo-Co acetate solutions were deposited on the substrates by dip-coating using solutions of Mo(CH_3_COO)_2_ (0.04% p/v) and Co(CH_3_COO)_2_·4H_2_O (0.02% p/v) salts in ethyl alcohol. Both solutions were stirred for 2 h and kept in the dark to prevent photodecomposition. The dip-coating was performed in two steps. In a first step, the substrates are immersed in Mo (II) acetate solution, and then calcined in an oven at 673 K for 20 min. After that, the substrates are cooled to room temperature and the dip-coating process is repeated using the Co (II) acetate solution. The substrates are then calcined again in an oven at 673 K for 20 min. The result of this process of impregnation and subsequent calcination is the elimination of organic compounds and the oxidation of metals. Both oxides will subsequently be reduced to their metallic form during the SWNT growth process. It is assumed that the Mo acts by avoiding the aggregation of Co nanoparticles, which are the real catalyst for the growth of the SWNTs [37]. Figure 1 shows the different stages of the substrate preparation process.

### 2.2. Contact Angle Measurements and Surface Free Energy Calculations of the Different Materials

In order to determine the factors that influence the selective deposition of catalysts on different substrates, wettability and Surface Free Energies (SFEs) studies were carried out. The SFE was calculated from contact angle measurements using an optical contact angle analyzer (OCA 15plus, Dataphysics, GmbH, Filderstadt, Germany) by the sessile drop method. Ultrapure water (Milli-Q, 18.2 MΩ cm), diiodomethane (Aldrich Chemistry, Madrid, Spain 99.0% purity) and ethylene glycol (Panreac Química S.A., Madrid, Spain, 99.5% purity) were employed as wetting agents. Using the syringe pump, a drop with a volume of 1 μL was deposited on the surface of the substrate at a constant rate. The data were recorded using a high-speed framing camera within 20 s after scaffold contact. The droplet shape was determined by two main methods, namely the ellipse and Young-Laplace techniques, and the contact angle was measured using the SCA20 software of the OCA 15plus system, with an accuracy of ±0.1°.

From the contact angle measurements, the SFE was calculated by the Owens, Wendt, Rabel, and Kaelble (OWRK) method [38,39,40]. According to this model, the impregnation of a support with a liquid is mainly determined by the difference in surface energy between the liquid (in our case, the catalyst solution) and the substrate, as expressed by Young’s equation:

(1) $$\gamma_{S}=\gamma_{SL}+\gamma_{L}\;cos\theta ,$$

where *γ_S_* is the SFE of the substrate, *γ_SL_* is the surface tension of the substrate-liquid, *γ_L_* is the surface tension of the liquid, and *θ* is the contact angle.

According to the OWRK model, the surface tension of the solid and liquid phases can be divided into the sum of two components (polar and dispersive):

(2) $$\gamma_{S}=\gamma_{S}^{P}+\gamma_{S}^{D}$$

(3) $$\gamma_{L}=\gamma_{L}^{P}+\gamma_{L}^{D}$$

By combining Equations (1)–(3), the following equation can be obtained:

(4) $$\gamma_{SL}=\gamma_{S}+\gamma_{L}-2\left[{\left(\gamma_{L}^{P}\gamma_{S}^{P}\right)}^{1/2}+{\left(\gamma_{L}^{D}\gamma_{S}^{D}\right)}^{1/2}\right]$$

which can be rearranged to obtain the following expression:

(5) $$\left(1+cos\theta \right)\gamma_{L}/2{\left(\gamma_{L}^{D}\right)}^{1/2}={\left(\gamma_{S}^{P}\right)}^{1/2}{\left(\gamma_{L}^{P}/\gamma_{L}^{D}\right)}^{1/2}+{\left(\gamma_{S}^{D}\right)}^{1/2}$$

which corresponds to the equation of a line, where ${\left(\gamma_{S}^{P}\right)}^{1/2}$ is the slope, and ${\left(\gamma_{S}^{D}\right)}^{1/2}$ is the intercept.

According to Equation (5), from the linear regression of the measured angles, it is possible to obtain the surface free energy of the analyzed substrates.

**Figure 2:**
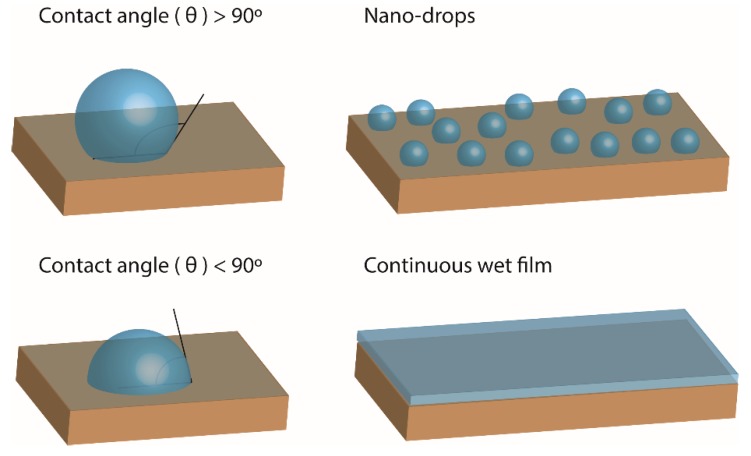
Schematic of the influence of surface free energy on the arrangement of the catalyst solution during the dip-coating process.

Young’s Equation (1) can be rewritten as:

(6) $$cos\theta =(\gamma_{S}-\gamma_{SL})/\gamma_{L}$$

so that when $\gamma_{SL}$ is greater than $\gamma_{S}$, cosθ<0, and so θ>90 (see Figure 2). This situation increases the dewetting of the liquid on the substrate, favoring the appearance of small drops. The presence of these drops can be related to a greater mobility of the catalyst on the surface of the substrate, which would imply the possibility of the coalescence of the catalyst nanoparticles during the thermal treatment, producing much larger particles, with dimensions unsuitable for the growth of SWNTs. On the other hand, if the angle formed between the surface of the substrate and the drop of the liquid containing the catalyst is less than 90° (see Figure 2), the liquid will cover the substrate in a homogeneous manner, increasing the wettability. In this sense, better wettability can inhibit the mobility of the catalyst particles and, thus, hinter particle coalescence [41].

From a practical point of view, the OWRK model requires us to calculate the contact angles of at least two liquids (three are recommended) on the surface to be studied: a liquid with a strong polar component (water), another with a strong dispersive component (diiodomethane) and an intermediate one (ethylene glycol). The SCA21 software was used to perform the adjustment of points to a line, providing the SFE. For each liquid and substrate, around 450 values of contact angles were collected.

### 2.3. Growth of Vertically Aligned SWNTs

The synthesis of SWNTs was carried out by using an AC-CVD system at atmospheric pressure. The substrates, whose synthesis was described above, were placed on a quartz boat within the tubular CVD reactor (25 mm inner diameter, 1000 mm length). Next, the system was closed and samples were exposed to a mixture of argon (75%) and hydrogen (25%), at a flow rate of 320 sccm. Simultaneously, the reactor was heated up to 1073 K, using a temperature ramp of 30 K min^−1^. The substrates were held at 1073 K for 10 min. During this thermal treatment, the Mo and Co oxides are transformed into metallic nanoparticles of a certain diameter, which will allow the growth of SWNTs. Then, the reaction conditions were readjusted, keeping the hydrogen and argon flows constant at their initial levels, and adding to this mixture the carbon source. In our case, ethanol was used and a nitrogen flow was passed through a flask containing a mixture of ethanol:water (99.5:0.5 *v*/*v*). The flow of nitrogen, saturated with the ethanol-water mixture, was sent to the reactor at a rate of 500 sccm. The optimal growth time of SWNTs was established at ca. 20 min. To complete the synthesis process, the heating of the oven was interrupted. After removing the stream of hydrogen, and nitrogen saturated with ethanol, a flow of Ar (300 sccm) was introduced into the reactor until the system reached room temperature.

### 2.4. Characterization Methods

Field emission scanning electron microscopy (FEG, Philips XL-30S, Eindhoven, the Netherlands) at 20 kV, was used to study the morphology, alignment, and main characteristics of the as-synthesized SWNTs. High resolution transmission electron microscopy (HRTEM, JEOL 300 KV, Peabody, MA, USA) was used to determine the wall structure of the individual carbon nanotubes.

The Raman spectra were measured at room temperature using a confocal Raman Microscope (Witec alpha300R, Witec, Ulm, Germany), equipped with a 532 nm Nd:YAG laser, a Leica microscope, and an electrically refrigerated CCD camera. Raman spectra, obtained in the range of 100–3700 cm^−1^, were collected at a spectral resolution of 4 cm^−1^, using a laser power of ca. 7 mW to minimize the heating of the samples. In all cases, a scanning area of 25 × 25 μm was used, with Raman images of 50 × 50 pixels, and integration times per pixel of 1.2 s. The collected Raman spectra were analyzed using the Witec Control Plus software.

X-ray photoelectron spectroscopy (XPS) studies were carried out by means of a Perkin-Elmer PHI 3027 spectrometer (Waltham, MA, USA), equipped with a double-pass cylindrical mirror analyzer, using the MgKα (1253.6 eV) radiation of a twin anode in the constant analyzer energy mode with a pass energy of 50 eV. The pressure of the analysis chamber was always below 10^−9^ mbar. The binding energy and the Auger kinetic energy scale were regulated by setting the C 1s transition at 284.6 eV.

Atomic force microscopy (AFM) measurements were carried out using a Nanotec SPM system, operating in tapping mode at room temperature and in ambient air conditions.

### 2.5. Field Emission Measurements

**Figure 3:**
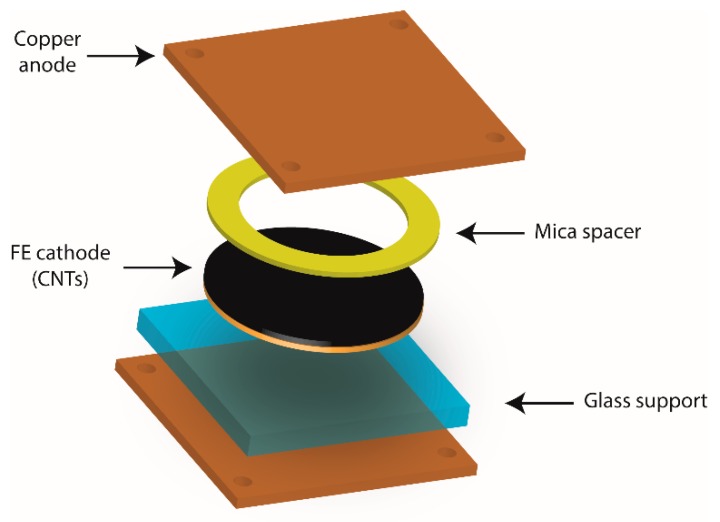
Schematic of the field emission device used in this research.

The field emission measurements from the synthesized SWNTs were carried out at 10^−7^ mbar, using the assembly shown in Figure 3. The diode configuration of the field-emission device is formed by a glass support and a piece of Kapton (75 µm thick), to completely isolate the sample of SWNTs from the vacuum system. The sample was fixed to the device using conductive double-sided carbon tape and, to exactly select the emission area, a mica sheet having a 2 mm diameter perforation was used. The mica sheet, with a thickness of 25 μm, also acts as a good insulator to prevent cathode-anode contact in the device. The cathode consisted of SWNTs grown on the micro-patterned sample of TiN-Si. The measurement areas were approximately π mm^2^, but the efficient emission area is considerably smaller due to the selective growth of SWNTs on the micro-patterned sample, and also to areas where there was no growth. In fact, as a result of the dimensions of the grid used as a mask, the SWNTs covered less than 50% of the area analyzed. A polished copper rod located above the SWNT sample was used as the anode. The anode voltage was applied using a high-voltage source unit (100–1000 V), equipped with a current limiter. An ammeter (Keithely 197) was connected in series to collect the produced field emission and a voltmeter, in parallel with the source, to accurately measure the applied voltage. The emission current (with μA sensitivity) was measured under various applied voltages, ranging from 100 V to 1000 V. To analyze the data, the emission current density, J (mA cm^−2^), versus electric field, E (V µm^−1^), as well as the Fowler-Nordheim (F-N) plot (ln (J/E^2^) versus 1/E) were plotted [42].

## 3. Results

### 3.1. Deposition of the Catalysts by Dip-Coating

**Figure 4:**
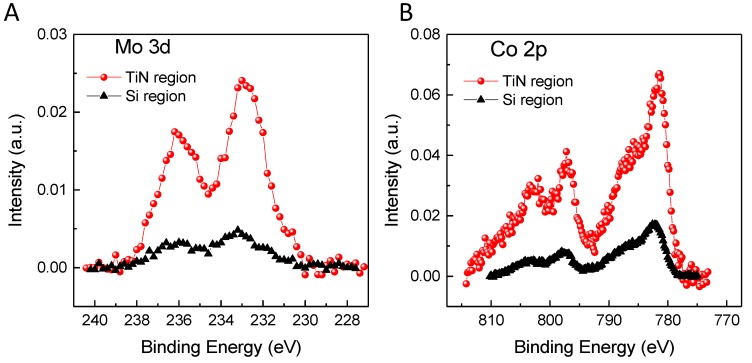
XPS spectra of Mo 3d (**A**) and Co 2p (**B**) on TiN and sputtered-Si regions, after catalyst deposition and subsequent calcinations to 673 K.

The results of the catalyst deposition by dip-coating were obtained by XPS. Figure 4 shows the spectra of Mo 3d and Co 2p measured on both surfaces, after the deposition and calcination of the catalyst. The Mo 3d spectra (Figure 4A) show two components at ca. 233 eV and 236.2 eV, which have been assigned to the Mo 3d_5/2,3/2_ spin-orbit components, respectively, of Mo^+6^ species [43]. As observed, the intensity of both peaks is much greater when measured on the TiN surface than on the sputtered-Si surface, which indicates that most of the Mo oxide nanoparticles are located on the surface of TiN. Figure 4B shows the XPS spectra of Co 2p when analyzing the two surfaces considered. Co 2p has two main components at ca. 781 eV and 797 eV, assigned to Co 2p_3/2,1/2_ spin-orbit components, possibly from a mixture of Co^2+^ and Co^3+^ from the presence of Co_3_O_4_ [44]. This assignment is also justified by the presence of shake-up satellites at ca. 788 eV and 803 eV [44,45]. The results shown in the case of cobalt are totally comparable to those observed for molybdenum. The surface of TiN shows the highest peak intensities compared to the surface of sputtered-Si, indicating that the catalyst is deposited mainly on the surface of TiN.

**Figure 5:**
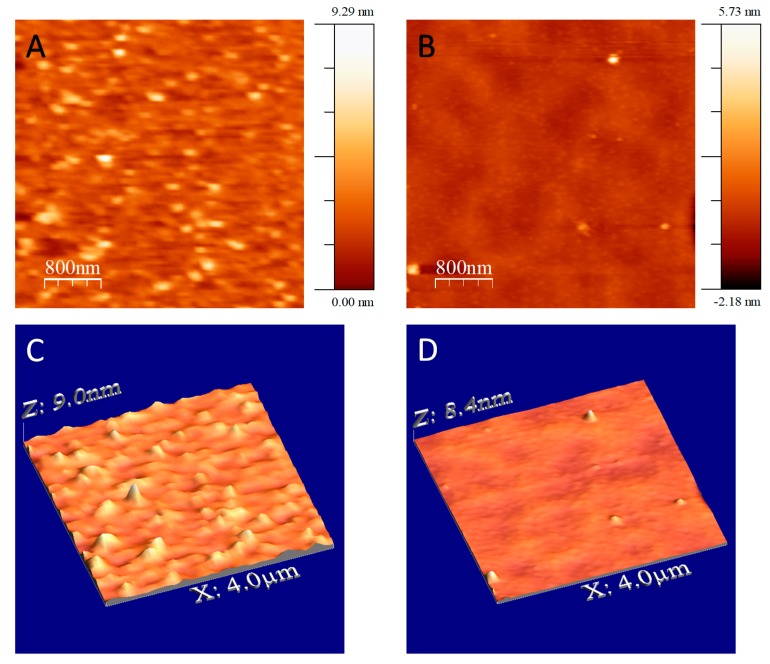
AFM images of native-SiO_2_ (**A**,**C**) and sputtered-Si (**B**,**D**) substrates after the deposition of the catalyst and heating to 673 K.

An AFM study was also carried out of the surfaces of TiN, native-SiO_2_ and sputtered-Si, after the process of impregnation of the catalyst by dip-coating, and just before the growth of the carbon nanotubes. In this case, as previously reported [36], the roughness of the TiN substrate after the thermal treatment with CVD was so high that it was not possible to observe the presence of catalyst nanoparticles. Therefore, by means of AFM only the surface of Si substrates could be analyzed. Figure 5 shows both surfaces (native-SiO_2_ and sputtered-Si) after the deposition of the catalyst, and after heating up to 673 K, to detect the formation of nanoparticles. The average particle size of the catalysts (as oxides) was estimated to be ca. 100 nm. As observed, the roughness of the two surfaces after the deposition of the catalyst is very different. The mean square roughness (RMS) measured by AFM of the region corresponding to native-SiO_2_ (Figure 5A,C) is higher (0.8 nm) than that presented by the surface of sputtered-Si (0.3 nm, Figure 5B,D), which indicates that the catalyst is deposited on the surface of native-SiO_2_, and not on the sputtered-Si. The average height along the *Z*-axis changes considerably from one surface to another. Thus, as can be seen, on the surface of Si before being sputtered (native-SiO_2_), the height of the particles reaches ca. 9 nm, compared to the surface of sputtered-Si, which shows a residual height of approximately 0.4 nm due to the possible presence of few catalyst residues.

To explain the observations made by AFM and XPS, and to understand the causes of this behavior, surface free energy measurements of several substrates, including TiN and sputtered-Si, were carried out. For this, and as already described above, the OWRK model was used, based on the measurements of the contact angle of liquids deposited on solid surfaces, as shown in Figure 2. The contact angles of three different liquids were measured: deionized water, with a predominant polar component over the dispersive component, ethylene glycol characterized by having a dispersive component slightly larger than the polar component, and diiodomethane with a predominant dispersive component [46]. The contact angles measured for the three test liquids on different substrates are listed in Table 1.

From the experimentally measured contact angles, the SFE could be estimated for each of the substrates considered. The thermodynamics of surfaces states that, in equilibrium, a system composed of solid components will rearrange to the lowest surface free energy; therefore, any material will tend to remain on the surface of the substrate having a higher SFE, increasing the stability of the system. In our case, the substrates used were TiN and sputtered-Si. The SFE values of both materials are not very different, showing a difference of only 1 mJ m^−2^, which is enough to confirm that the catalyst nanoparticles are deposited predominantly on the surface of TiN and not on sputtered-Si. These results agree with a previous work by our group [36], in which when the surfaces of TiN and native-SiO_2_ alternate, the deposition of the catalysts occurs selectively on the native-SiO_2_ and not on TiN, as expected according to the results shown in Table 1.

### 3.2. Growth of SWNTs

**Figure 6:**
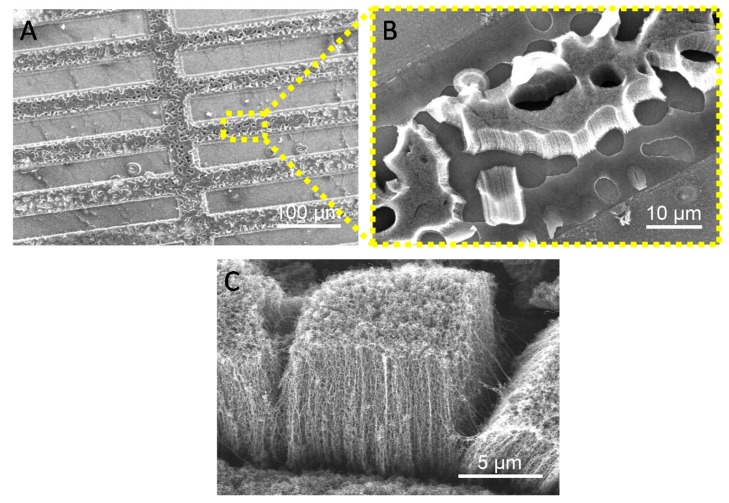
SEM image of the patterned sample after the AC-CVD synthesis (**A**). Zoom-in corresponding to the TiN lanes where VA-SWNTs are grown (**B**,**C**).

After the deposition of catalysts on the substrates (with patterned areas of sputtered-Si and TiN), SWNTs were grown by AC-CVD, as described in Section 2.3. Figure 6 shows FESEM images at different magnification of the sample, containing alternating rectangular regions of TiN (200 nm thick) and sputtered-Si, after the synthesis procedure. A dense growth of VA-SWNTs on the TiN regions is clearly observed. As seen in Figure 6A, the growth of VA-SWNTs is limited to the zones corresponding to TiN, with no growth observed on the surface of sputtered-Si. Figure 6B,C show a homogenous growth of densely packed and vertically aligned SWNTs, with an average height of ca. 8 μm. This selective growth of SWNTs, exclusively along the TiN lanes, agrees with the XPS, AFM, and SFE studies explained above.

Interestingly, and possibly due to the thermal treatment used for the growth of SWNTs, the surface of TiN is no longer homogeneous, showing small zones of sputtered-Si in areas that should correspond to TiN (see Figure 6B). As can be seen, in these areas no growth has occurred, which could hinder the possible application of these materials, at least using, as in this case, thin TiN coatings of 200 nm. In this regard, growth tests are currently being carried out using much higher TiN thicknesses that could avoid this effect.

In addition, it should be noted that the growth of SWNTs does not occur over large areas of TiN when they are not in the vicinity of sputtered-Si surfaces. This effect has only been observed in the case of substrates based on TiN and sputtered-Si, and not when the substrate shows TiN zones adjacent to SiO_2_ areas. In the latter case, the growth occurs only on SiO_2_ [36], regardless of whether there are TiN zones in the vicinity. A plausible explanation for this effect is the small difference in SFE. When there are contiguous areas of TiN and sputtered-Si, the catalyst tends to be located on the surface of TiN. However, when the areas of TiN are sufficiently large, preferential deposition on TiN versus sputtered-Si is no longer effective.

**Figure 7:**
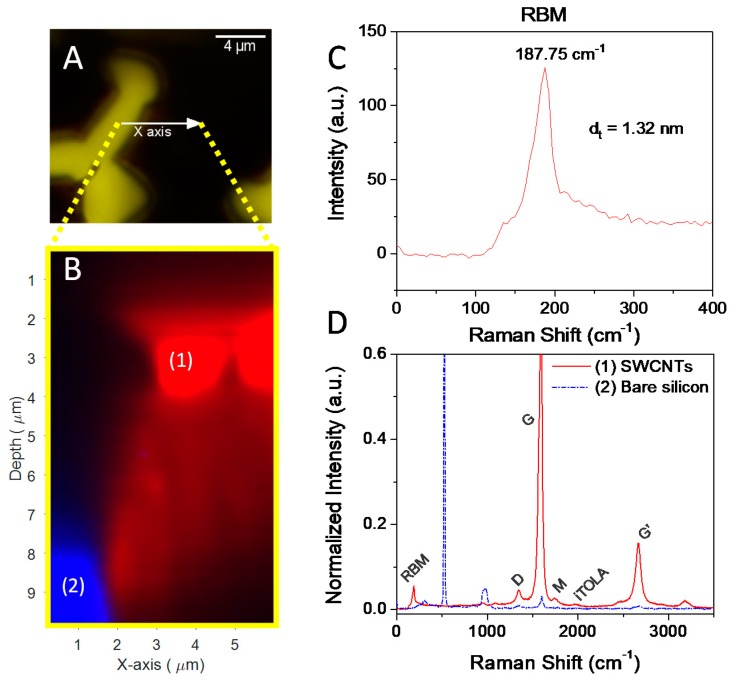
Optical image of the synthesized SWNTs (arrow indicates the analyzed Raman area, and the yellow and black zones correspond to silicon and carbon nanotubes, respectively) (**A**); Raman image analysis performed at different depths of the sample (**B**); Raman spectrum corresponding to the RBM signal (**C**); and Raman spectra measured in zones marked as (1) and (2) in the blue and red zones of (**B**,**D**).

The synthesized SWNTs were also characterized by Raman spectroscopy, using a 532 nm laser source. The sample analyzed was the same as that shown in Figure 6, with carbon nanotubes grown on TiN lanes. Figure 7 shows the optical image of the analyzed region, together with the corresponding Raman spectra. The Raman system used, Witec alpha300R, allowed the development of Raman tomographic imaging experiments, obtaining Raman information along the depth of the sample, on the *Z*-axis. Thus, along the *X*-axis (arrow in Figure 7A), which corresponds to a distance of 6 μm, Raman analyses were performed at different depths of the sample (Figure 7B), with a maximum depth of 8 μm, which corresponds to the length of the synthesized SWNTs. This study allowed us to observe the complete absence of nanotubes in the region of sputtered-Si (blue in Figure 7B), unlike in the TiN region, which showed the presence of densely packed SWNTs (red in Figure 7B).

**Figure 8:**
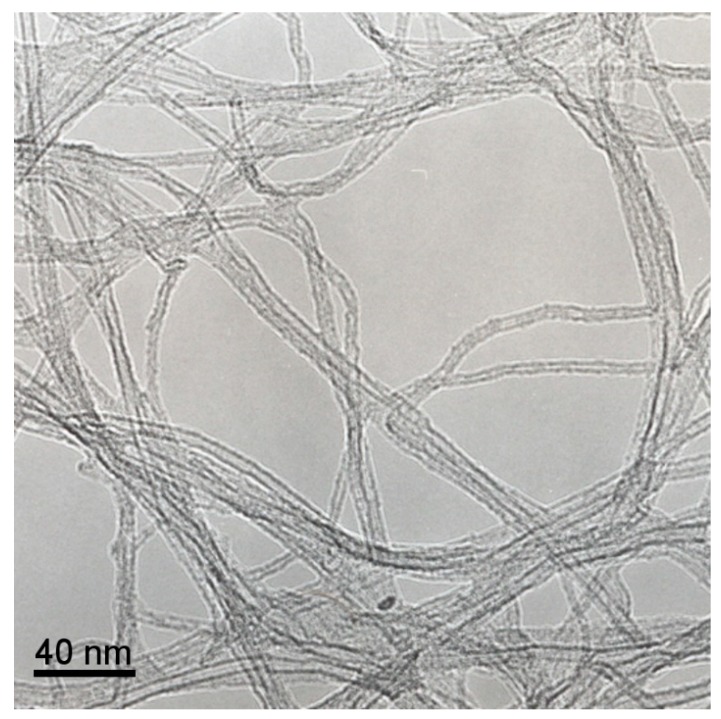
TEM image of SWNTs selectively grown on TiN surface.

Near the surface, the Raman spectrum (red line, Figure 7D) shows the typical characteristics of SWNTs, with the G band at 1594 cm^−1^ and the D band at 1340 cm^−1^. The high ratio of intensities of the G band to the D band (G/D ratio) indicates the purity of the nanotubes, possibly with low density of defects [47]. Moreover, the Radial Breathing Modes (RBMs) signal appears at frequencies lower than 300 cm^−1^, indicating that the nanotubes are SWNTs. Figure 7C shows an expansion of the Raman spectrum in this region. The Raman shift of the different RBMs correlates with the diameter of the SWNTs, by using the empirical formula *d* = 248/*ν_B_* [48], where *ν_B_* is the corresponding Raman shift (cm^−1^) and *d* is the diameter of the SWCNT (nm). The diameter determined by this approach shows a narrow distribution at ca. 1.32 nm. This value is clearly lower than that determined by HRTEM (see Figure 8), with diameters ranging from 3 to 5 nm. These results are indicative that SWNTs show some inhomogeneity and dispersion of diameters. Nevertheless, to the best of our knowledge, this is the first time that vertically-aligned SWNT forests have been grown on TiN surfaces by dip-coating and CVD.

As one of the potential applications of these densely packed SWNTs, their behavior as field emission cathodes was studied. Field emission properties of the SWNT forests grown on TiN substrates were evaluated using the diode configuration, described in Section 2.3 and schematized in Figure 3, in a high vacuum chamber with a base pressure of ca. 10^−7^ mbar. According to this setup, the maximum emission area corresponds to the area of the circular perforation made in the mica sheet (π mm^2^), and the maximum distance between the cathode (SWNTs) and the anode is approximately 17 μm (difference between the mica thickness and the maximum length of the SWNTs).

**Figure 9:**
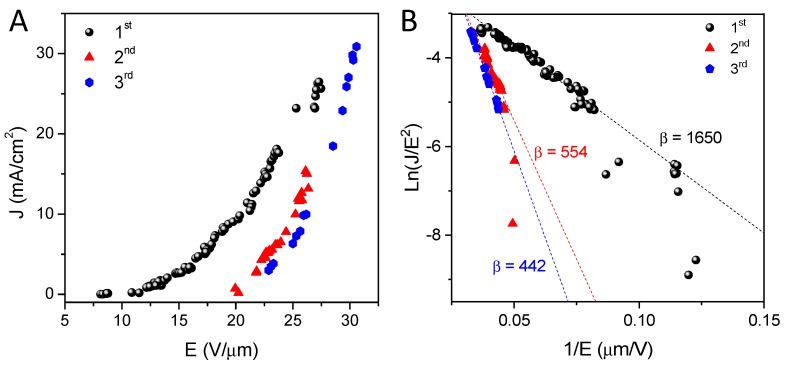
Field emission performance of SWNTs grown on TiN. J-E curves (**A**) and Fowler-Nordheim plots (**B**) of three consecutive field emission measurements on SWNTs samples.

Figure 9A illustrates the current density versus electric field (J-E curves) of the SWNTs emitters after three consecutive tests, and the corresponding Fowler-Nordheim (F-N) plots are given in Figure 9B.

The field enhancement factor (β) represents the ratio of the local electric field around the emitter tips of SWNTs and the applied electric field, and can be determined from the F-N plot, by means of the following equation [49]:

(7) ln(JE2)=a− 6.8 × 103 ∅32βE,

where ∅ is the carbon nanotube work function (eV), and the units of J and E are mA cm^−2^ and V µm^−1^, respectively.

It was assumed that the work function of the SWNTs was 4.7 eV [50]. After the first measurement test, the estimated field enhancement factor (β) was ca. 1650. This value is significantly high, and in agreement with those that in the literature [51,52,53]. During the second and third consecutive measurements, a reduction of β was observed up to values close to 500, which could be attributed to the partial degradation of the sample by Joule effect, as well as to the appearance of problems with electrical contacts. This behavior could be related to the fact that in the first emission test only carbon nanotubes that have the longest length, and that are closer to the collector, emit; once they have evaporated (by the Joule effect), the nanotubes of shorter lengths (possibly more numerous) begin to emit, increasing the emission surface. Another possible explanation could be that the nanotubes of smaller diameter are those that present an emission at a lower E (V/μm). After the first cycle, if these have been degraded by the Joule effect, the larger-diameter nanotubes are those that start emitting, and so on. In addition, and during the different repetitions of these experiments, it was observed that the vacuum of the chamber was degraded to values of ca. 10^−6^ mbar, probably due to the desorption of gas molecules adsorbed on the SWNTs walls, which has the effect of reducing the emission current.

The first results shown in this research point to the fact that SWNTs grown directly on TiN conductive substrates are an excellent option to develop Field Emission devices, so at present we are working to obtain much more stable devices with better performance.

## 4. Conclusions

In this work, we report on the selective synthesis of SWNTs by AC-CVD on TiN conductive substrates, and their characterization. We started with Si (100) substrates that were coated with a TiN thin film of 200 nm. These substrates were subsequently sputtered with Ar^+^ to generate alternating lanes of TiN and sputtered-Si. The substrates were then subjected to impregnation of the catalyst by dip-coating. The results reveal that densely packed SWNT forests grow selectively over TiN conductive areas, and not over Si. This behavior has been attributed to the difference between the SFEs of the TiN and sputtered-Si, and to the preferential deposition of the catalyst on the surface of TiN. The as-synthesized SWNTs were additionally tested as field emission cathodes.

## Figures and Tables

**Table 1:** Average values of contact angles of three solvents in different substrates, and SFE values determined for each substrate.

Substrate	θ (Water)	θ (Diiodomethane)	θ (Ethylene Glycol)	SFE ^1^ (mJ m^−2^)
Thermal SiO_2_	22.0°	47.2°	25.7°	64.7
Native-SiO_2_	43.9°	43.3°	21.3°	56.8
TiO_2_	52.7°	45.9°	45.8°	48.1
TiN	61.6°	-	48.2°	40.6
Sputtered-Si	69.7°	45.5°	37.0°	39.6

^1^ Surface Free Energy.
